# Isolation, Characterization, and Medicinal Potential of Polysaccharides of *Morchella esculenta*

**DOI:** 10.3390/molecules26051459

**Published:** 2021-03-08

**Authors:** Syed Lal Badshah, Anila Riaz, Akhtar Muhammad, Gülsen Tel Çayan, Fatih Çayan, Mehmet Emin Duru, Nasir Ahmad, Abdul-Hamid Emwas, Mariusz Jaremko

**Affiliations:** 1 Department of Chemistry, Islamia College University Peshawar, Peshawar 25120, Pakistan; riaz94awan@hotmail.com (A.R.); drakhtarmuhammad@icp.edu.pk (A.M.); nasirshah121@yahoo.com (N.A.); 2 Department of Chemistry and Chemical Processing Technologies, Muğla Vocational School, Muğla Sıtkı Koçman University, 48000 Muğla, Turkey; gulsen_tel@hotmail.com (G.T.Ç.); fatihcayan@mu.edu.tr (F.Ç.); eminduru@mu.edu.tr (M.E.D.); 3 Core Labs, King Abdullah University of Science and Technology (KAUST), Thuwal 23955-6900, Saudi Arabia; abdelhamid.emwas@kaust.edu.sa; 4 Division of Biological and Environmental Sciences and Engineering (BESE), King Abdullah University of Science and Technology (KAUST), Thuwal 23955-6900, Saudi Arabia

**Keywords:** *Morchella esculenta*, polysaccharopeptides, anticholinesterase activity, antioxidant, tyrosinase inhibition

## Abstract

Mushroom polysaccharides are active medicinal compounds that possess immune-modulatory and anticancer properties. Currently, the mushroom polysaccharides krestin, lentinan, and polysaccharopeptides are used as anticancer drugs. They are an unexplored source of natural products with huge potential in both the medicinal and nutraceutical industries. The northern parts of Pakistan have a rich biodiversity of mushrooms that grow during different seasons of the year. Here we selected an edible *Morchella esculenta* (true morels) of the Ascomycota group for polysaccharide isolation and characterization. Polysaccharopeptides and polysaccharides from this mushroom were isolated using the green chemistry, hot water treatment method. Fourier transform infrared spectroscopy revealed the sugar nature and possible beta-glucan type structure of these polysaccharides. Antioxidant assays showed that the deproteinized polysaccharides have moderate free radical scavenging activity. These isolated polysaccharides exhibited good acetylcholinesterase (AChE) and butyryl cholinesterase (BChE) inhibition activities. Therefore, these polysaccharides may be valuable for the treatment of Alzheimer’s and Parkinson’s diseases. Further bioassays are needed to discover the true potential of *M. esculenta* polysaccharides for medicinal purposes.

## 1. Introduction

Since prehistoric times, and across different civilizations, mushrooms have been utilized as a dietary source and in medication [1]. Mushrooms possess bioactive constituents that are antioxidant, anti-inflammatory, immunomodulatory, and antidiabetic [2,3,4,5]. There are several mushrooms whose polysaccharides have been well characterized by biophysical and biochemical techniques, and their bioactivities show promise for different diseases [4,5]. Among these, *Ganoderma*, *Trametes* or *Coriolus* (commonly known as Turkey tail), *Lentinus*, *Morchella*, and several other genera have displayed both nutritional and medicinal properties [6]. The *Pleurotus* mushroom genus is one of the cultivated types, and are generally called oyster or tree mushrooms [7]. The polysaccharides and proteins of *Pleurotus* possess anticancer and antiviral properties [7]. Immunomodulation and anticancer activity are some of the unique properties that the mushroom polysaccharides are known for, which has caused an increase in their consumption as food over the past few decades [8,9,10]. Mushroom polysaccharides contain various types of glycosidic bonds and are thus grouped as beta-glucans, alpha-glucans, and heteroglycans [9]. Some of them interact with proteins to form polysaccharopeptides [9]. So far, three important clinically well-established antitumor polysaccharides, including lentinan and protein-containing polysaccharides like krestin, have been isolated from *Lentinus* species, *Schizophyllum,* and Turkey tails [9,11,12]. These mushrooms have quite large commercial markets in East Asian countries [9,11,12]. Chemical analyses of lentinan and schizophyllan showed that they are complete β-glucans, while the polysaccharide Krestin (PSK) is a protein-bound beta-glucan [9,11,12]. A polysaccharopeptide obtained from a strain of the *Coriolus* species in China also had anticancer and immune-boosting properties [9]. In Japan, for cancer treatment, a few grams of PSK is given orally to patients during chemotherapy [4]. Different biological test results of PSK showed improvement of immune functions, antiviral defense, body regulation of cholesterol, and prebiotic activity [4,13]. Although the exact mode of action of these mushroom polysaccharides has not yet been established, using them as additional adjuvants with cancer drugs may be of value in cancer treatment. The *Agaricus bisporus* β-glucans interact with intestinal cells and activate the immune system and enterocytes [14]. Several other species of *Agaricus* mushroom have been noted to contain antioxidants and have anticancer properties [15]. Because of these beneficial roles of the mushroom polysaccharides, they have become an interesting area of study in medicinal chemistry. Here, we selected *Morchella esculenta* mushrooms for polysaccharide isolation and purification, which were then tested for specific bioactivities.

The *Morchella* genus is one of the most favored mushrooms, and as such, it is highly priced [16]. The *Morchella esculenta* contains all the important nutrients, from carbohydrates, proteins, polyunsaturated fatty acids, secondary metabolites like phenolic compounds, etc. [17]. The methanolic extract from the mushrooms has potent antioxidant properties and antibacterial activities against different bacteria [17]. It has been observed that the utilization of *Morchella esculenta* polysaccharides as a food in mice increased the useful gut microbiota as well as the short-chain fatty acids in the body [18]. A 43.6 kDa purified polysaccharide from *M. esculenta* was shown to contain glucose, mannose, galactose, and arabinose as the monomer units [19]. An 81 kDa *M. esculenta* polysaccharide was shown to have the potential to cease the growth and spread of human colon cancer in HT29 cells, and its potency is dose and time dependent [2]. Several sterols and fatty acids recognized in the methanolic extract of *M. esculenta* fruiting bodies had antitumor activity when tested against different human lung cancer cell lines with IC_50_ values in the range of 157 to 278 μM [20]. A polysaccharide isolated from the mycelium of *M. esculenta* showed antiproliferative activity against hepatoma cell lines [21]. A heteropolysaccharide isolated from *M. esculenta* had anti-melanogenesis properties [22]. These properties were investigated both in vitro (B16F10 melanoma cells) and in vivo (zebrafish larvae), showing a reduction of melanin production without any cytotoxicity [22]. Thus, *M. esculenta* polysaccharides can be used in skin cancer treatment. The galactomannan polysaccharide isolated from *Morchella esculenta* enhances the immune response to different diseases [16] and modulates the immune system [23]. It has been reported that polysaccharides from *Morchella conica* can help treat hepatocarcinomas by reducing the generation of free radicals [21]. It has also been demonstrated that polysaccharides from *Morchella conica* can potentially modulate the immune system by inhibiting nitric oxide production in lipopolysaccharide-treated macrophages; thus, these polysaccharides could be used for treating inflammatory diseases [24]. The partial chemical modification of polysaccharides from *Morchella angusticepes* Peck, by adding a carboxymethyl group at C6 in its sugars, resulted in a reduction in cholesterol levels in rats [25]. Thus, these polysaccharides can potentially be used to lower blood and liver cholesterol levels and may be valuable for treating heart coronary diseases. The aim of this scientific work was to determine the free radical scavenging and anticholinesterase and tyrosinase properties of wild-grown *M. esculenta* polysaccharides in proteinized and deproteinized forms.

## 2. Materials and Methods

### 2.1. Mushroom Collection and Preliminary Treatment

*Morchella esculenta* mushrooms were collected from the natural habitat of the khyber Pakhtunkhwa region of Pakistan. A mycological survey was performed by a local expert from the botany department of our university, and a mushroom sample was deposited in their herbarium. The mushrooms were washed with distilled water to remove soil debris, then shade dried and crushed through a grinder. Volatile and non-volatile organic pigments and compounds were removed by dipping the samples in methanol for two weeks, and the process was repeated three times [26,27]. The samples were then filtered and the residue left on the filter paper was further used for polysaccharide extraction [26,27].

### 2.2. Polysaccharide Extraction

The residue left on the filter paper was dried at room temperature. The polysaccharides were extracted by hot water extraction in which the sample was heated in distilled water at 80 °C for 1–2 h [26,27]. To remove any particulate matter from the solution, it was filtered three times. Then, ethanol was added to the filtrate in a 1:3 ratio. The obtained solution was centrifuged at 3000× *g* for 25 min [26,27]. After centrifugation, the upper layer of supernatant was discarded, while the pellet of proteinated polysaccharides was separated, washed with ethanol three times, dried, and stored at 0 °C in the freezer [26,27].

### 2.3. Deproteinization of Polysaccharides

Deionized water was added to the proteinized polysaccharides, and the pH of this solution was brought to pH 3.0 using 0.1 M of HCl solution [26,27]. The diluted and proteinized sample was left at room temperature for 1 day [26,27]. After this, it was centrifuged, and this time the supernatant was isolated in a china dish and oven dried [26,27]. The layer of polysaccharides collected were labelled as the deproteinized polysaccharides [26,27].

### 2.4. Fourier Transform Infrared Spectroscopy

The Fourier transform infrared spectroscopy (FTIR) spectra of polysaccharides were collected using the Tensor II Bruker Germany FTIR spectrometer using the attenuated total reflection (ATR) sampling method. This spectrophotometer was equipped with a global source, a KBr beam and a deuterated triglycine sulphate detector. Spectra were collected from 400 to 4000 cm^−1^. Approximately 0.5 mg of the sample was used for each test.

### 2.5. Antioxidant Bioassays

#### 2.5.1. 2,2-diphenyl-1-picrylhydrazyl (DPPH) Assay

The first free radical scavenging activity was performed with 2,2-diphenyl-1-picrylhydrazyl (DPPH) reagent [28]. The reaction mechanism contained the absorption spectrum of free radical-generated changes when they were reduced by an antioxidant compound, and was recorded at 517 nm, where DPPH had maximum absorption [28,29]. Approximately 40.0 μL of sample solutions of different concentrations of polysaccharides were taken, and to these solutions, 160 μL of 0.4 mM DPPH methanolic solution was added. The absorbance was recorded at 517 nm after 30 min of incubation. Using Equation (1), the free radical scavenging activity of the mushroom polysaccharides was computed [29,30] as:

(1) $$I\;\left(\%\right)=\frac{A_{control}-A_{sample}}{A_{control}}\times 100.$$

#### 2.5.2. 2,2′-azinobis-(3-ethylbenzothiazoline-6-sulfonic acid) (ABTS^•+^) Cation Assay

The ABTS^•+^ was generated by reacting 7 mM ABTS solution of H_2_O and 2.45 mM potassium persulfate, and incubated for 12 h in the dark at 25 °C [29,31]. Before starting the experiment, the ABTS^•+^ solution was diluted with ethanol so that the absorbance was 0.708 ± 0.025 at a wavelength of 734 nm [29,31]. A 160 µL solution of ABTS^•+^ was added to 40 µL of the sample solution in ethanol at different concentration ranges. After 10 min, the percent inhibition at 734 nm was calculated for each concentration compared to the blank (ethanol) absorbance values [29,31,32]. The antioxidant potential was calculated according to Equation (1) [29,31,32].

#### 2.5.3. Cupric Reducing Antioxidant Capacity (CUPRAC) Assay

The chromogenic redox compound utilized in this assay was a bis(neocuproine) copper(II) chelate [33,34]. At pH 7.0 of the solution, the absorbance of the Cu(I)-chelate, which is due to the redox reaction with the reducing testing agent, was measured at 450 nm [33,34]. The color was due to the formation of Cu(I)-NC chelation. All the reaction conditions were at optimum levels [33,34]. Slight modifications were made to this assay for the *Morchella* polysaccharides [26,29]. In a multiwell plate, 50 µL of 10 Mm Cu (II) solution, 50 µL of 7.5 mM neocuproine, and 60 µL of ammonium acetate (NH_4_Ac) of 1 M solutions at pH 7.0 were added. To make the total volume 200 µL, 40 µL of polysaccharides of different concentration series were added to the respective wells on the plate [26,29]. After 60 min of incubation, the absorbance change in the test and blank samples was measured at 450 nm [26,29].

### 2.6. Enzyme Inhibitory Activities

#### 2.6.1. Anti-Acetylcholinesterase Activity

The modified Ellman method was used for this assay using acetylcholinesterase (AChE) from electric eels and AChE iodide as a substrate [26,29,35]. Additionally, 5,5′-Dithio-bis(2-nitrobenzoic) acid (DTNB) was utilized for cholinesterase action [26,29]. Then, 130 µL of 100 mM sodium phosphate buffer (pH 8.0), 10 µL of polysaccharide solution made in ethanol in a series of different concentrations, and 20 µL of AChE solution in buffer were mixed and incubated for 15 min at room temperature, and 20 µL of 0.5 mM DTNB was added to them [26,29]. The reaction was then started by the addition of 20 µL of 0.71 mM of acetylthiocholine iodide [26,29]. The hydrolysis of the substrate was monitored at 412 nm by the formation of a yellow color 5-thio-2-nitrobenzoate anion that appears when DTNB reacts with thiocholine [26,29].

#### 2.6.2. Butyryl Cholinesterase Inhibition Assay

The butyryl cholinesterase (BChE) inhibition assay was performed using BChE from horse serum (BChE, EC 3.1.1.8, 11.4 U/mg, Sigma, St. Louis, MO, USA), and butyryl thiocholine iodide as its substrate, respectively, in a colorimetric analysis [36]. In total, 10 μL of polysaccharide solution made in 0.2% DMSO, 79 μL of 20 mM sodium phosphate buffer (pH 7.6), and 1 μL of prepared enzymes (with final concentrations of 0.035 unit/mL for BChE, and final concentrations of 1 to 500/1000 μM for the compounds tested) were combined and incubated for 15 min [26,29,37]. To the combined solutions, 10 μL of substrate solution was added (final concentration of 4 mM for butyryl thiocholine iodide) and incubated for 30 min [26,29,37]. The reaction was halted by adding 900 μL of DTNB-phosphate-ethanol reagent and the absorbance was measured quickly at 412 nm on a microplate reader [26,29,37]. The percent inhibition at 50% (IC_50_) was calculated with galanthamine as a positive control [26,29,37].

#### 2.6.3. Anti-Tyrosinase Assay

Anti-tyrosinase activity was observed spectrophotometrically, as established by Masuda with some changes [38]. The tyrosinase enzyme from mushrooms was used with I-3,4-dihydroxyphenylalanine (I-DOPA) as a substrate in this reaction [26,29,38]. As such, 150 µL of the 100 mM sodium phosphate buffer (pH 6.8), 10 µL of polysaccharide solution made in ethanol in a series of different concentrations, and 20 µL of the tyrosinase solution in buffer were combined and placed for 10 min at 37 °C, and 20 µL L-DOPA was supplemented to them [26,29,38]. The sample and blank absorbances were recorded at 475 nm using a 96-well microplate [26,29,38].

#### 2.6.4. Statistical Analysis

The results obtained during different bioassays were the mean of the triplicate samples. The data were recorded as the mean, and the standard deviation was calculated for them. The Student’s test was used here to get the main variation between the means, and *p* values < 0.05 were considered as significant.

## 3. Results and Discussion

### 3.1. Polysaccharide Extraction

The yield of the crude proteinized polysaccharide and deproteinized polysaccharides from *M. esculenta* fruiting bodies varied every time during the extraction process. These crude polysaccharides were a mixture of polysaccharides that varied in molecular weight. The average yield of the crude proteinized polysaccharides was 3%, while that of deproteinized polysaccharides was 1.3%, from the dried powdered fruiting body taken during each extraction experiment. The loss in yield occurred during various steps of the experiment that included the hot water extraction, ethanol washing steps, collection after drying in the oven, and deproteinization steps. Optimization of the extraction method can be useful for getting a better yield.

### 3.2. FTIR Analysis

**Figure 1:**
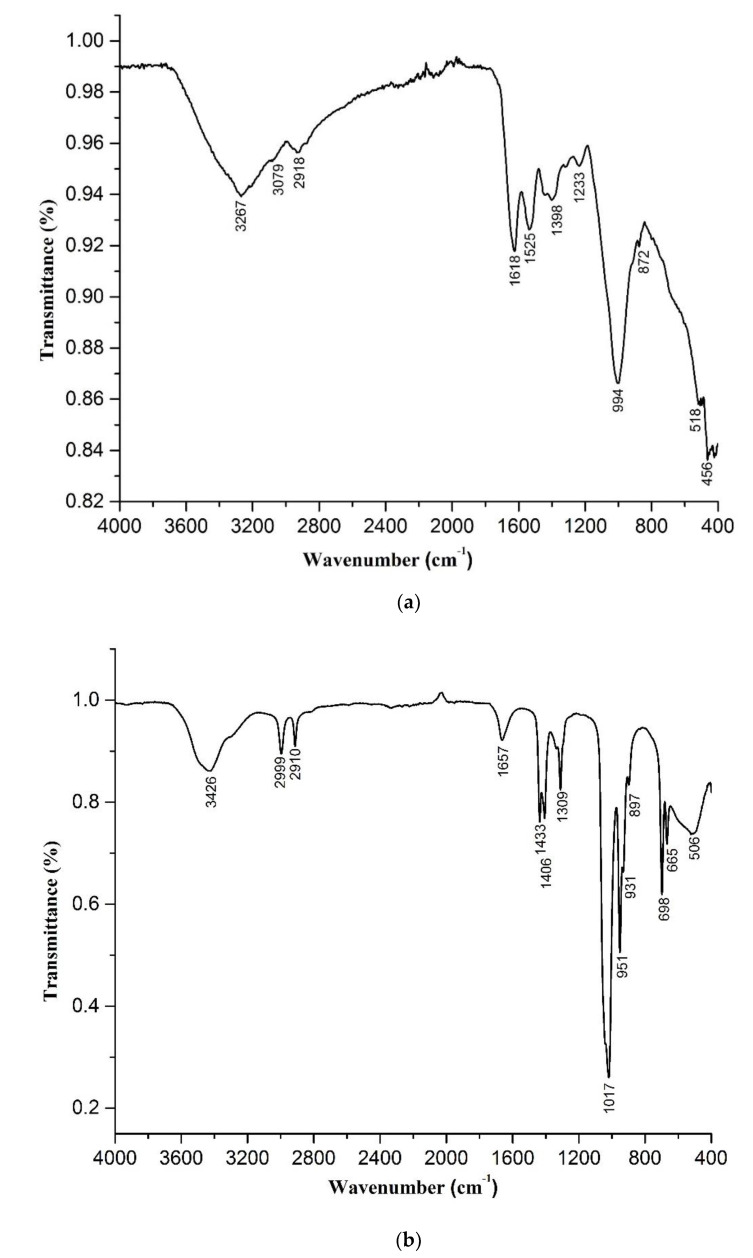
(**a**) Fourier transform infrared spectroscopy (FTIR) spectrum of proteinized forms of polysaccharides of *M. esculenta.* (**b**) FTIR spectrum of deproteinized forms of polysaccharides of *M. esculenta.*

The FTIR spectra of proteinized and deproteinized *M. esculenta* crude polysaccharides are presented in Figure 1a,b, respectively. In Figure 1a, the peaks at 3267 cm^−1^ in the proteinized polysaccharides, and 3427 cm^−1^ in the deproteinized polysaccharides FTIR spectrum of *M. esculenta*, revealed the existence of hydroxyl groups [39]. These bands come from the stretching of the hydroxyl groups. The band at 2918 cm^−l^ in the spectrum of proteinized polysaccharides, and at 2999 cm^−l^ and 2910 cm^−l^ in the spectrum of deproteinized crude polysaccharides of *M. esculenta,* were representative of the -CH stretching vibration band showing the presence of the polysaccharide. N–H groups present in the proteinized polysaccharides of *M. esculenta* caused peaks at a frequency from 1618 cm^−l^ to 1525 cm^−1^. The bands’ intensity for amide groups decreased for deproteinized polysaccharides because of the removal of proteins. However, a similar band at 1640 cm^−1^ represented the C=O group of ester carbonyls studied by Li et al. [40]. In another study, a 1616 cm^−1^ band was also assigned to the C=O group to represent the presence of uronic acid in *M. esculenta* polysaccharides [2]. The carbonyl group band appeared at 1657 cm^−l^ in the deproteinized polysaccharides. The removal of proteins caused a decreased in the intensity of both N–H and C=O groups in the deproteinized polysaccharides. The 1398 cm^−1^ peak can be assigned to the presence of galactans, as previously reported for the 1400 cm^−1^ peak in the *M. esculenta* crude polysaccharides [2]. Similar bands at 1433 and 1406 cm^−1^, representing galactans, were also present in the deproteinized polysaccharides (Figure 1b).The large peak at 1003.15 cm^−1^ was almost similar to the 1050 cm^−1^ peak reported by Li et al. for the -C–O group [40]. The shoulder at 875.33 cm^−1^ can be assigned to the β-glycosidic bond. This β-glycosidic bond peak was also reported at 890 cm^−1^ by other authors [40]. It has been suggested that this β-glycosidic linkage plays a role in the antitumor and immunomodulatory functions of the *Morchella* polysaccharides [40]. The small shoulder from 600 to 400 cm^−1^ is usually that of the pyranose structure in the polysaccharides. It was reported earlier from C^13^-NMR that *M. esculenta* polysaccharides are rich in glucose, mannose, and galactose, while small amounts of arabinose, rhamnose, and xylose are also present, and arranged through 1–3 and 1–4 β-linkages [40].

From the deproteinized polysaccharide FTIR spectrum (Figure 1b), several new features could be observed, and several peak intensities were enhanced compared to the proteinized polysaccharide spectrum. At 1664.01 cm^−1^, a small shoulder revealed the existence of carbon–carbon double bonds (C=C) of deproteinized polysaccharides of *M. esculenta.* The peaks in the FTIR spectrum of the deproteinized crude polysaccharides of *M. esculenta* at 1436 cm^−l^ to 1310 cm^−l^ represented the bending vibrations of the O–H groups. The high intensity bands located between 1320 cm^−l^ and 1003 cm^−l^ in the proteinized crude polysaccharides from *M. esculenta* depict the pyranose rings, and the region of 1019 cm^−l^ in the monosaccharide also showed similar peaks. The main bands in the spectrum of proteinized crude polysaccharides centered at 875 cm^−1^, 898 cm^−l^, and 898.21 cm^−1^, and the deproteinized crude polysaccharides from *M. esculenta* indicated the presence of β-type linked glycosidic bonds.

### 3.3. Antioxidant Activity

The consumption of antioxidant-containing fruits and vegetables is important for living organisms, and particularly for humans, as antioxidants defend the body from destruction by reactive oxygen species (ROS). Excessive generation of ROS and imbalanced metabolism can lead to a condition of oxidative stress, resulting in severe impairments and disorders like Alzheimer’s disease, Parkinson’s disease, and several other diseases due to the damage of proteins, DNA, and other biomolecules. The antioxidant properties of secondary metabolites, extracted through hot water and ethanolic extract from mushroom species, for example *Boletus*, *Agaricus,* and *Agrocybe,* have been well described. These mushrooms are used in different foodstuffs as a flavoring ingredient. Three different antioxidant assays were performed to test the antioxidant potential of the isolated polysaccharides from *M. esculenta*. These antioxidant assays included the DPPH, ABTS, and CUPRAC assays.

#### 3.3.1. DPPH Assay

In the DPPH bioassay, the radical hunting power of the proteinized and deproteinized polysaccharides from *M. esculenta* was evaluated and compared with the reference compounds α-tocopherol and butylated hydroxyanisole (BHA). The DPPH assay revealed that the radical scavenging activity of the deproteinized polysaccharides of these wild-collected morels contained moderate antioxidant activity that was concentration dependent. In contrast, the proteinized polysaccharides from *M. esculenta* had no antioxidant activity at any of the tested concentrations. The deproteinized polysaccharide samples of *M. esculenta* displayed potent antioxidant activity and the trend of antioxidant potential was concentration dependent, as shown in Table 1. The IC_50_ value of deproteinized polysaccharides of *Morchella esculenta* was 282.95 μg·mL^−1^, compared to the reference compounds BHA and α-tocopherols, which were 37.20 ± 0.41 and 19.80 ± 0.36 μg·mL^−1^, respectively. The lower values of IC_50_ obtained here for the deproteinized polysaccharides compared to the standard BHA and tocopherol are due to the large molecular weight of these biopolymers, the hydrogen addition or removal strength, and the different electron donating and accepting properties. Different glucans isolated from *Lentinula edodes* (Shiitake mushrooms) have an IC_50_ of 183.8 μg/mL, which is almost similar to the antioxidant values we reported here for *M. esculenta* [41]. Xiong et al. reported DPPH activity with an EC_50_ value of 1.18 ± 0.04 mg/mL for crude polysaccharides from *M. importuna,* which was lower than the standard compound [42].

#### 3.3.2. ABTS ^•+^ Assay

The ABTS bioassay is a useful method for defining the antioxidant potential of H-atom donating and chain-breaking antioxidants. The ABTS antioxidant activity of the isolated polysaccharides was tested and compared with the reference antioxidants BHA and α-tocopherol. The IC_50_ values of some polysaccharides were near the range of the reference standards, while some polysaccharides showed weak activity, as their percent inhibition was lower than the standards, and their IC_50_ values were higher than the reference standard compounds. Table 1 indicates that the morels’ proteinized polysaccharides were slightly active at various concentrations. The deproteinized polysaccharides of *M. esculenta* displayed moderate antioxidant activity in comparison with the proteinized polysaccharides, as presented in Table 1. In terms of the IC_50_ value, the deproteinized polysaccharides of *M. esculenta* was 130.69 μg·mL^−1^. In comparison, the reference standard compounds BHA and α-tocopherol showed IC_50_ values of 38.51 and 11.82 μg·mL^−1^, respectively. The reported *M. esculenta* mycelium hot water-ethanolic extract had a IC_50_ value of 87.50 ± 4.33 and this extract possibly contained flavonoids, phenols, and other small organic molecules [43].

#### 3.3.3. CUPRAC Assay

The CUPRAC antioxidant assay of the mushroom polysaccharides was performed and compared with the reference compounds BHA and α-tocopherol. The CUPRAC assay showed that the antioxidant potential of the mushroom polysaccharides was dependent on its concentration, increasing with increasing concentration. The reference compounds BHA and α-tocopherol had absorbances at 0.5 (A_0.5_) of 66.72 and 24.40 µg/mL, respectively. In Table 1, the CUPRAC assay results demonstrate that the polysaccharides in both forms had reasonable free radical scavenging activities, in contrast to BHA and α-Tocopherol. The A_0.5_ values of the deproteinized samples were better than that of the proteinized crude polysaccharides. Thus, the deproteinized samples were more active in the CUPRAC assay. A similar moderate antioxidant activity was also observed in *Morchella* mushrooms from China, together with immune-enhancing properties [44]. An exopolysaccharide from a submerged culture of *M. esculenta* showed potent antioxidant activities in an in vivo experiment [45]. The antioxidant and neuroprotective roles of crude polysaccharides from *M. importuna* have also been observed previously, and it was suggested that they can enhance the function of radical scavenging enzymes in the body [42].

### 3.4. Enzyme Inhibition Assays

#### 3.4.1. Acetylcholinesterase (AChE) Inhibitory Activity

Acetylcholinesterase functions by breaking acetylcholine and other choline esters used as neurotransmitters, hence inhibition of this enzyme prolongs the lifetime of these neurotransmitters [46]. In Alzheimer’s disease, the expression of this enzyme increases, and the hydrolysis rate of AChE also increases [47]. Data collected for the proteinized and deproteinized polysaccharides of the *M. esculenta* mushroom are presented in Table 2. Galanthamine was used as a reference standard in the enzyme inhibition assay for acetylcholinesterase. The proteinized polysaccharides of *M. esculenta* showed better blocking of the enzyme compared to the deproteinized polysaccharides. The IC_50_ values of both samples of *M. esculenta* were higher than 100 μg·mL^−1^ as compared to the standard galanthamine, which had an IC_50_ of 5.0 ± 0.13 μg·mL^−1^ (Table 2). These data show the proteinized polysaccharides of *M. esculenta* were low to moderately potent for blocking AChE function when compared to the standard galanthamine. We can therefore infer that Morchella polysaccharides have therapeutic potential and could be used in conjugation with other drugs to control Alzheimer’s disease and other related neurological diseases.

#### 3.4.2. Butyryl Cholinesterase Inhibition Assay

BChE is another common and broad cholinesterase that uses choline esters as its substrates [26]. Inside the human body, BChE exists in organs including the liver, and also in blood plasma. We performed the BChE assay using galanthamine as a reference compound [26]. Blockers of cholinesterase from different mushrooms are undiscovered therapeutic agents that could be used for the treatment of neurodegenerative diseases, and likely have vast medicinal abilities [26]. The proteinized polysaccharides of *M. esculenta* were weakly inhibitive when used in smaller amounts, while they were more potent at higher concentrations (Table 2). The deproteinized sample of *M. esculenta* displayed higher potency. The IC_50_ value of the proteinized polysaccharides of *M. esculenta* was 128.62 µg·mL^−1^ and the value of the deproteinized samples was 28.74 µg·mL^−1^, while that of the standard galanthamine was IC_50_ 11.55 ± 0.93 µg·mL^−1^. The lower inhibitory power of the polysaccharides compared to the standard compound might be due to their binding capacity, depending on the size of the active pocket of the enzyme and the interaction between the enzyme and the tested inhibitor. Although the galanthamine has two-fold higher butyryl cholinesterase inhibition than the deproteinized polysaccharide, both these polysaccharides are suitable bionutrients due to lower toxic nature.

#### 3.4.3. Tyrosinase Enzyme Inhibition

We also tested the isolated polysaccharides for their tyrosinase enzyme inhibition activity using kojic acid as a reference standard. Table 3 shows that the *M. esculenta* mushroom proteinized polysaccharides had no activity at the initial concentrations, but were weakly inhibitive at higher concentrations. The deproteinized polysaccharides of *M. esculenta* also showed no repression at the initial concentrations; however, weak repression was observed at 100 µg/mL. The IC_50_ values for the *M. esculenta* proteinized and deproteinized polysaccharides were above 100 µg·mL^−1^, while that of the standard kojic acid was 8.25 ± 0.36 µg·mL^−1^. These higher IC_50_ values for the polysaccharides of *M. esculenta* indicated that they did not interact with the binding pocket of the tyrosinase enzymes as compared to the kojic acid, which may be because of their higher molecular weight, or that they cannot induced any changes in the enzyme when they interacted with it. These results showed that the *M. esculenta* polysaccharides do not have any effect on skin pigmentation and melatonin metabolism.

## 4. Conclusions

Polysaccharides, particularly β-glucans, strengthen the immune system and are found in high levels in mushrooms. Therefore, many mushroom species are both a valuable dietary source as well as a possible therapeutic source for treating prevalent disorders like cancer, inflammation, and diabetes. The preliminary outcome of this research showed that the crude polysaccharides of the selected mushroom species *M. esculenta* had notable antioxidant potential, specifically the deproteinized crude polysaccharides. Thus, this mushroom genus is a suitable source of antioxidants and anticancer agents. Based on the state-of-the-art knowledge about the antioxidant potential, the enzyme inhibitory test against acetylcholinesterase and butyryl cholinesterase enzymes, the fruiting bodies, and the polysaccharides and other secondary metabolites present in them, mushrooms are potentially useful agents for the treatment of Alzheimer’s disease and other related neurological diseases. The regular consumption of this mushroom may also help in the enhancement of cognition abilities and memory in old age and provide a good nutrition. Moreover, the further investigation of the properties of other metabolites present within the discussed mushroom species needs to be undertaken in order to fully characterize their value as nutrients for humans.

## Figures and Tables

**Table 1:** 2,2-diphenyl-1-picrylhydrazyl (DPPH), 2,2′-azinobis-(3-ethylbenzothiazoline-6-sulfonic acid) (ABTS) cation and cupric reducing antioxidant capacity (CUPRAC) free radical scavenging activity of polysaccharides of *M. esculenta.*

**DPPH Assay**					
**Polysaccharides Sample**	**50 µg** **/mL**	**100 µg** **/mL**	**200 µg** **/mL**	**400 µg** **/mL**	**IC_50_ (µg/mL)**
Proteinized	NA	NA	NA	NA	NA
Deproteinized	7.88 ± 1.87	21.87 ± 0.90	40.96 ± 1.68	66.92 ± 2.58	282.95
BHA	79.37 ± 0.33	86.21 ± 0.16	87.13 ± 0.09	87.27 ± 0.03	37.20 ± 0.41
α-Tocopherol	66.68 ± 0.43	87.28 ± 0.13	87.14 ± 0.28	87.44 ± 0.09	19.80 ± 0.36
**ABTS Cation Assay**					
**Polysaccharides Sample**	**50 µg** **/mL**	**100 µg** **/mL**	**200 µg** **/mL**	**400 µg** **/mL**	**IC_50_ µg/mL**
Proteinized	4.71 ± 0.50	7.37 ± 0.72	10.89 ± 2.87	19.32 ± 2.14	>400
Deproteinized	25.78 ± 1.53	41.32 ± 1.09	69.60 ± 1.07	82.96 ± 2.23	130.69
BHA	90.79 ± 0.19	91.02 ± 0.05	91.50 ± 0.20	91.18 ± 0.26	38.51 ± 0.54
α-Tocopherol	66.52 ± 3.77	88.80 ± 2.57	91.95 ± 0.09	91.86 ± 0.12	11.82 ± 0.09
**CUPRAC Assay**					
**Polysaccharides Sample**	**50 µg/mL**	**100 µg/mL**	**200 µg/mL**	**400 µg/mL**	**A_0.50_ µg/mL**
Proteinized	0.12 ± 0.01	0.14 ± 0.01	0.22 ± 0.04	0.30 ± 0.03	>400
Deproteinized	0.18 ± 0.02	0.28 ± 0.03	0.46 ± 0.04	0.84 ± 0.03	215.79
BHA	0.95 ± 0.11	1.52 ± 0.11	2.47 ± 0.01	3.59 ± 0.07	66.72 ± 0.81
α-Tocopherol	0.35 ± 0.11	0.54 ± 0.17	0.85 ± 0.02	1.51 ± 0.04	24.40 ± 0.69

**Table 2:** Acetylcholinesterase (AChE) and butyryl cholinesterase (BChE) inhibitory assay of polysaccharides of *M. esculenta***.**

**Acetylcholinesterase Inhibition Assay**					
**Polysaccharides Samples**	**12.5 µg/mL**	**25 µg/mL**	**50 µg/mL**	**100 µg/mL**	**IC_50_ µg/mL**
Proteinized	18.72 ± 2.17	28.68 ± 2.13	33.38 ± 2.57	39.67 ± 2.11	>100
Deproteinized	2.64 ± 1.44	3.51 ± 2.12	17.45 ± 0.95	20.54 ± 0.50	>100
Galanthamine	55.31 ± 0.79	55.87 ± 0.97	61.66 ± 1.17	55.31 ± 0.79	5.0 ± 0.13
**Butyryl Cholinesterase Inhibition Assay**					
**Polysaccharides Samples**	**12.5 µg/mL**	**25 µg/mL**	**50 µg/mL**	**100 µg/mL**	**IC_50_ µg/mL**
Proteinized	14.96 ± 1.81	38.25 ± 1.96	45.11 ± 2.08	48.22 ± 2.28	128.62
Deproteinized	44.65 ± 2.66	49.61 ± 2.76	52.22 ± 2.75	54.08 ± 2.88	28.74
Galanthamine	50.88 ± 1.42	62.48 ± 0.02	66.47 ± 0.46	71.43 ± 0.06	11.55 ± 0.93

**Table 3:** Tyrosinase enzyme inhibition assay of polysaccharides from *M. esculenta*.

**Polysaccharides Samples**	**12.5 µg/mL**	**25 µg/mL**	**50 µg/mL**	**100 µg/mL**	**IC_50_ (µg/mL)**
Proteinized	NA	4.87 ± 0.89	16.12 ± 2.70	31.99 ± 2.32	>100
Deproteinized	NA	NA	NA	10.94 ± 1.55	>100
Kojic acid	50.49 ± 1.32	52.36 ± 0.81	56.98 ± 0.96	67.89 ± 1.05	8.25 ± 0.36

## Data Availability

Data will be provided upon request.
